# Research on Multiscale Characterization and Computational Modeling/Simulation of Metallic Materials

**DOI:** 10.3390/ma19071417

**Published:** 2026-04-02

**Authors:** Rui Wang, Jiaqing Li

**Affiliations:** 1 School of Mechanical, Materials, Mechatronic and Biomedical Engineering, University of Wollongong, Wollongong, NSW 2522, Australia; ruiw@uow.edu.au; 2 College of Chemical Engineering, Fuzhou University, Fuzhou 350108, China

Metallic materials are fundamental to strategic sectors such as aerospace and energy systems. Understanding the cross-scale relationship between microstructure and macroscopic service performance has long been a central issue in materials science. With the rapid development of multiscale characterization techniques and computational modeling and simulation, the traditional trial-and-error paradigm in metallic materials development is being progressively transformed. A systematic research framework has emerged, covering atomic-scale defect evolution [1,2,3,4], mesoscale microstructural evolution [5,6,7], macroscopic mechanical performance [8,9,10], and artificial intelligence-assisted materials design [11,12,13,14]. These advances provide strong support for the development of high-performance metallic materials, process optimization, and service reliability evaluation. This Special Issue (SI)—entitled “Multiscale Characterization and Computational Modeling/Simulation of Metallic Materials”—presents recent advances in theoretical analysis, experimental characterization, and computational simulation of metallic materials. This editorial summarizes the fifteen contributions included in the SI.

Macroscopic mechanical properties remain key indicators for evaluating the engineering performance of metallic materials. These properties are typically characterized using experimental techniques such as in situ slow strain rate tensile testing [15,16,17] and room-temperature compression testing [18], which enable the quantitative evaluation of strength, ductility, and strain-hardening behavior [19]. Zhang et al. [20] conducted eighteen single-factor experiments and showed that discontinuous scanning tracks and lack-of-fusion defects occurred when laser power was below 800 W or the defocus distance reached −5 mm, while scanning speed was identified as the most critical parameter influencing thermal history. Based on upsetting and hot rolling experiments, Cojocaru et al. [21] reported that increasing deformation temperature or impact energy significantly enhanced the deformation degree, whereas deformation resistance and mechanical work decreased. Xu et al. [22] investigated hydrogen embrittlement using electrochemical cathodic hydrogen charging combined with slow strain rate tensile testing and revealed a non-monotonic temperature dependence, with 308 K identified as a critical threshold.

The macroscopic behavior of metallic materials originates from mesoscale microstructural characteristics, making microstructural characterization essential for understanding the underlying mechanisms. Advanced techniques, including scanning electron microscopy (SEM), electron backscatter diffraction (EBSD), X-ray diffraction (XRD), and energy-dispersive spectroscopy (EDS) [23,24,25,26], enable detailed analyses of grain refinement, grain boundary evolution, phase transformation, second-phase distribution, and texture development [27,28,29]. Ni et al. [30], using EBSD and microhardness measurements, demonstrated that focused high-pressure torsion (HPT) deformation refined grains through repeated elongation and fragmentation processes, accompanied by a nonlinear increase in microhardness. Wan et al. [31], through OM, SEM/EDS, and XRD analyses, observed that Ca suppressed grain growth and promoted the transformation of the β-Mg_17_Al_12_ phase into a Ca- and Sn-containing blocky structure distributed along grain boundaries. Xiang et al. [32] reported that spark plasma sintering completely decomposed TiO_2_, allowing oxygen atoms to dissolve into the Ti-Zr matrix. Xu et al. [33] observed using metallographic optical microscopy that the phase compositions of ten types of bcc steels consisted of ferrite and pearlite, martensite, or spheroidal cementite. Hardness tests further showed that microhardness decreased with increasing indentation depth, which was consistent with the indentation size effect.

From atomic interactions to macroscopic engineering behavior, cross-scale computational modeling and simulation [34,35,36,37,38] have become powerful tools for revealing constitutive mechanisms and predicting material performance. Zheng et al. [39], using molecular dynamics simulations, investigated irradiation-induced defect evolution in single-crystal and bicrystal tungsten under different temperatures and primary knock-on atom (PKA) energies. The results showed that grain boundaries hindered the recombination of interstitial atoms and vacancies, while vacancies in bicrystals tended to form larger clusters. Wang et al. [40] employed finite element simulations and demonstrated that a 10–15 nm austenite layer improved fracture toughness by altering crack propagation paths and delaying crack growth, whereas crack orientation had only a minor influence. Tian et al. [41] simulated the leakage and diffusion behavior of buried pure hydrogen pipelines using ANSYS Fluent 2023R2. Their results clarified that hydrogen migration was restricted when the leakage aperture was ≤2 mm, while the hazardous zone expanded nonlinearly when the pressure exceeded 2 MPa. An SQP optimization prediction model was also constructed, achieving a mean absolute error below 10%. Tian et al. [42] conducted transient numerical simulations of hydrogen–methane mixing in T-type pipelines using ANSYS Fluent. They found that increasing the hydrogen proportion shortened the mixing distance but prolonged the total mixing time, whereas increasing the methane flow rate reduced the overall mixing time but required a longer pipeline for complete mixing.

Microstructural evolution and failure processes in metallic materials are essentially macroscopic manifestations of coupled atomic-, mesoscale-, and macroscale interactions under multiple physical fields [43,44,45]. Consequently, characterization or modeling at a single scale often cannot fully reveal the intrinsic relationships among composition, structure, and performance. Integrated multiscale experimental and computational approaches are therefore essential. Trusov et al. [46] developed a multiscale recrystallization model demonstrating that sub-grain coalescence acted as a dynamic recovery mechanism and competed energetically with recrystallization nucleation. Focusing on corrosion in magnesium alloys, Horstemeyer et al. [47] established a quantitative model incorporating multiple corrosion mechanisms based on internal state variable theory and multiscale modeling concepts, enabling corrosion behavior to be predicted directly from elemental composition.

With the rapid development of materials data science, artificial intelligence and machine learning [48,49] have emerged as promising approaches for addressing the long development cycles and high costs associated with conventional materials research. Essa et al. [50] proposed an artificial neural network (ANN) model with a 2-8-4 architecture capable of reliably predicting the mechanical properties of friction stir welded AA5754-H24 aluminum alloy joints, achieving higher prediction accuracy for tensile strength, elongation, and weld nugget zone hardness than for thermo-mechanically affected zone hardness. Román-Sedano et al. [51] developed an ANN-based predictive model using experimentally derived variables and analyzed the relative importance of the inputs using the Garson algorithm, achieving correlation coefficients of 0.96 and 0.80 for predicting the effective diffusion coefficient and steady-state flux, respectively.

With continued methodological advances, future research on metallic materials will increasingly emphasize the integration of multiscale experiments, computational simulations, and data-driven methods. On the one hand, multiscale characterization techniques are expected to evolve toward in situ and dynamic capabilities. Techniques such as in situ SEM, TEM, and in situ XRD thermodynamic analysis [52,53] will enable real-time observation of microstructural evolution during service. On the other hand, high-fidelity cross-scale coupling of physical models should be strengthened by integrating computational frameworks such as density functional theory, molecular dynamics, and crystal plasticity finite element methods [54,55], enabling more accurate prediction of material performance throughout the service lifecycle. In addition, deeper integration between physics-based modeling and machine learning should be promoted, particularly in emerging areas such as physics-informed neural networks and machine-learning-based interatomic potentials [56,57].
